# Can a metric combining arm elevation and trapezius muscle activity predict neck/shoulder pain? A prospective cohort study in construction and healthcare

**DOI:** 10.1007/s00420-020-01610-w

**Published:** 2020-12-05

**Authors:** Suzanne Lerato Merkus, Svend Erik Mathiassen, Lars-Kristian Lunde, Markus Koch, Morten Wærsted, Mikael Forsman, Stein Knardahl, Kaj Bo Veiersted

**Affiliations:** 1 National Institute of Occupational Health, Oslo, Norway; 2 Centre for Musculoskeletal Research, Department of Occupational and Public Health Sciences, University of Gävle, Gävle, Sweden; 3 School of Engineering Sciences in Chemistry, Biotechnology and Health, KTH Royal Institute of Technology, Huddinge, Sweden; 4 IMM Institute of Environmental Medicine, Karolinska Institutet, Stockholm, Sweden

**Keywords:** Electromyography, Actigraphy, Neck/shoulder load, Physically demanding work, Musculoskeletal pain, Compositional data analysis

## Abstract

**Objective:**

To determine whether a composite metric of arm elevation and trapezius activity (i.e. neck/shoulder load) is more strongly associated with the 2-year course of neck and shoulder pain intensity (NSPi) among construction and healthcare workers than each exposure separately.

**Methods:**

Dominant arm elevation and upper trapezius muscle activity were estimated in construction and healthcare employees (*n* = 118) at baseline, using accelerometry and normalized surface electromyography (%MVE), respectively. At baseline and every 6 months for 2 years, workers reported NSPi (score 0–3). Compositions of working time were determined for arm elevation (< 30°; 30–60°;  > 60°), trapezius activity (< 0.5%; 0.5–7.0%; > 7.0%MVE), and a composite metric “neck/shoulder load” (restitution, low, medium, and high load). Associations between each of these three compositions and the 2-year course of NSPi were determined using linear mixed models.

**Results:**

Associations between exposure compositions and the course of NSPi were all weak and in general uncertain. Time spent in 0.5–7.0%MVE showed the largest and most certain association with changes in NSPi during follow-up (*β* = − 0.13; *p* = 0.037; corresponding to a −0.01 change in NPSi every 6 months). Among pain-free workers at baseline, medium (*β* = − 0.23; *p* = 0.039) and high (*β* = 0.15; *p* = 0.031) neck/shoulder load contributed the most to explaining changes in NSPi.

**Conclusion:**

The composite metric of neck/shoulder load did not show a stronger association with the course of NSPi than arm elevation or trapezius activity alone in the entire population, while some indications of a stronger association were found among those who were pain-free at baseline.

**Electronic supplementary material:**

The online version of this article (10.1007/s00420-020-01610-w) contains supplementary material, which is available to authorized users.

## Introduction

Neck and shoulder pain (NSP) occurs more often among employees in construction and healthcare than in most other occupations (Boschman et al. 2012; Davis and Kotowski 2015; Holmstrom and Engholm 2003; Occhionero et al. 2014). NSP has been associated with several biomechanical exposures that are prevalent in construction and healthcare (Mayer et al. 2012; Palmer and Smedley 2007; van der Molen et al. 2017). Assessment of such exposures have often been based on self-reports or observation; however, these methods are often inaccurate, and ‘true’ biomechanical exposures are better measured using objective assessments (Koch et al. 2016; van der Beek and Frings-Dresen 1998).

An association between NSP and work with elevated arms has been discussed in several reviews of an extensive scientific literature (Palmer and Smedley 2007; van der Molen et al. 2017). Most studies using self-reported arm elevation found positive associations with NSP, while studies using objective assessments show mixed results (Bodin et al. 2012; Bovenzi 2015; Coenen et al. 2016; Descatha et al. 2012; Hanvold et al. 2015; Koch et al. 2017; Nordander et al. 2016; Svendsen et al. 2004). The ambiguity among studies using objective assessments may, in part, result from the use of a traditional ‘threshold approach’, i.e. expressing exposure in terms of time above angles of elevation, thus ignoring how time is spent below that threshold (Pedisic et al. 2017). For example, the effect of the duration of arm elevation > 60° (“extreme” exposure) likely depends on whether time below 60° is spent between 30 and 60° (mild exposure) or below 30° (almost neutral posture). Ignoring the full composition of behaviours may result in misleading inferences (Dumuid et al. 2018). Compositional data analysis (CoDA) addresses this fallacy by acknowledging exposures forming parts of a whole as inter-related (Dumuid et al. 2018; Gupta et al. 2020; Pedisic et al. 2017). Thus, CoDA can, for instance, handle time spent in arm elevation > 60°, 30–60°, and < 30° as an inherently correlated set of exposures, and associate the whole set with an outcome of interest, such as pain.

Another biomechanical exposure at work that has been associated with pain in the neck and shoulders is upper trapezius muscle activity. A shorter duration of upper trapezius muscle ‘rest’ [i.e. activity < 0.5% of the maximal voluntary electrical activation (MVE)] (Thorn et al. 2007; Veiersted et al. 1993)), and a more frequent occurrence of periods of sustained muscle activity > 0.5%MVE, have both been associated with pain in the neck and shoulders (Hanvold et al. 2013; Ostensvik et al. 2009). However, these two exposures are complementary, i.e. compositional, and should be analysed as such.

Some studies have suggested that metrics combining multiple exposures, for instance, arm elevation and force use, into a ‘neck/shoulder load’ index are more strongly associated with NSP than an individual exposure (Garg et al. 2017; Palmer and Smedley 2007; van der Molen et al. 2017). So far, such composite metrics have mainly been based on self-reports or expert observation; one example being the Revised Strain Index (Garg et al. 2017; Moller et al. 2018; Palmer and Smedley 2007; van der Molen et al. 2017). However, a neck/shoulder load metric could also be constructed by combining different directly measured exposures, thus representing interactions between exposures in detail.

Using a CoDA approach, the present study among construction and healthcare workers aimed to determine whether a composite ‘neck/shoulder load’ metric developed by combining simultaneously recorded arm elevation and upper trapezius muscle activity is more strongly related to neck and shoulder pain over the course of two years than each of the two exposures separately.

## Methods

### Study design and population

For the purpose of determining associations between work environment factors and musculoskeletal disorders, we recruited construction and healthcare workers to participate in a longitudinal cohort study (Lunde et al. 2014). Employees were informed about the project in morning or lunch meetings; employees not able to attend received the information from their supervisors. At baseline, 594 construction and healthcare workers (response rate 51%) filled in a questionnaire (Lunde et al. 2014). Of these workers, 371 consented to participate in objective measurements, and 138 were selected based on availability and logistics, as well as their job title (to obtain a broad range of biomechanical exposures found in each sector). Pregnancy and cardiovascular disease were exclusion criteria (Lunde et al. 2014). Data collection commenced in the 1st quarter of 2014 and ended in the 1st quarter of 2017.

The study was approved by the Regional Committee for Medical and Health Research Ethics in Norway (2014/138/REK south east D). It was conducted in accordance with the Helsinki Declaration, and all participants gave written informed consent prior to the study.

### Objective exposure assessment

At baseline, upper arm elevation and upper trapezius muscle activity were monitored bilaterally for a full working day; only recordings from the dominant side were analysed in the present study. Ten muscle activity recordings were shorter than 4 h (between 1.4 and 3.6 h) due to technical problems with the EMG-equipment (*n* = 8), and due to premature termination of data collection, e.g. because equipment felt uncomfortable (*n* = 2). All ten recordings were considered representative, based on notes by the researchers on activities during the working day.

#### Upper arm elevation

The elevation angle of the upper arm was recorded using tri-axial accelerometers (Actigraph GT3X+, Actigraph, Florida, USA) placed 3 cm below the deltoid muscle insertion using double-sided tape, and covered with transparent film (Tegaderm, 3 M, St. Paul, Minnesota, USA). The *xyz*-coordinate system was defined prior to attaching the accelerometers to the upper arm by securing the actigraphs to a rail and placing this rail on a table top, first horizontally in two directions perpendicular to each other (the *x*- and *z*-axes), then vertically perpendicular to the table top (*y*-axis). The latter was used as the zero reference angle. The accelerometer raw data were extracted using ActiLife version 5.5 (Actigraph, Pensacola, FL, USA) and re-sampled to 10 Hz. The arm elevation angle throughout the measurement was then computed as the angle between the zero reference data vector and the measured data vector, using a custom-made program. Upper arm elevation was categorised in terms of duration (percentage of the working day) with the arm elevated < 30° (near neutral posture), 30–60° (mild elevation), and > 60° (extreme elevation).

#### Upper trapezius muscle activity

Upper trapezius muscle activity was recorded by bipolar surface electromyography (sEMG) using an ambulatory system (Mobi 8, TSMi, Enschede, the Netherlands). Cables connected the electrodes with a logger (240 g with batteries) placed between the shoulder blades (in a camelbak racebak shirt). The cables did not interfere with movements of the neck and shoulders. Self-adhesive pre-gelled Ag/AgCl electrodes (Ambu Neuroline 720, Ambu, Ballerup, Copenhagen) were placed according to recommendations in Mathiassen et al. (1995). The sEMG signal was amplified, band-pass filtered at 30–440 Hz, sampled at 1024 Hz, and controlled for movement artefacts and electromagnetic interference (Hansson et al. 1997, 2000). The signal was then root-mean-squared (RMS) converted in consecutive, non-overlapping epochs of 0.125 s, re-sampled to 10 Hz to synchronise with the arm elevation data, and normalised to an MVE (Korshoj et al. 2014; Mathiassen et al. 1995). The MVE was the largest 0.5 s moving window RMS value from three maximal voluntary isometric shoulder abductions, seated with arms elevated 90° in the scapular plane and fixed to a hanging scale placed just proximal to the elbow (Amagliani et al. 2010; Essendrop et al. 2001; Lunde et al. 2014; Mathiassen et al. 1995). Normalised sEMG was categorised in terms of percentages of the working day with activity < 0.5% MVE (“muscle rest”) (Nordander et al. 2000; Veiersted et al. 1993), 0.5–7.0% MVE (medium activity), and > 7.0% MVE (high activity) (Anton et al. 2003; Mathiassen and Winkel 1991).

#### Neck/shoulder load

A composite neck/shoulder load metric was developed from synchronized recordings of arm elevation and trapezius activity, categorised as the percentage of the working day at four exposure levels:

1. *Restitution*: trapezius activity < 0.5% MVE, irrespective of upper arm elevation
2. *Low load*: arm elevation < 30° with trapezius activity 0.5–7.0% MVE
3. *Medium load*: arm elevation < 30° with trapezius activity > 7.0% MVE, or arm elevation > 30° with trapezius activity 0.5–7.0% MVE
4. *High load*: arm elevation > 30° with trapezius activity > 7.0% MVE.

### Neck and shoulder pain

Pain intensity in the neck and in the dominant shoulder (NSPi) during the past four weeks was reported by the workers on a 4-point scale from 0 ‘no pain’ to 3 ‘severe pain’. One question considered pain in the neck and another considered pain in the dominant shoulder. The anatomical areas were illustrated by a mannequin (Kuorinka et al. 1987). NSPi, defined as the higher of the two scores, was recorded at baseline and every 6 months during the 2-year follow-up.

### Background information and confounders

At baseline, workers provided information on weekly working hours, age (years), seniority (years), gender (male/female), and pain duration in the neck and shoulders in the 12 months preceding baseline (on a 5-point scale ranging from never to daily). Additional health- and work related information was obtained both at baseline and every 6 months during the 2-year follow-up. This included height, weight, general health, control of work pacing, social climate, and self-reported arm elevation. Height and weight was used to calculate body mass index (BMI), which was classified into normal BMI (< 25 kg/m^2^) and overweight/obese (≥ 25 kg/m^2^). General health status was assessed using a single question from the SF-36 with responses from 1 ‘excellent’ to 5 ‘poor’ (Ware 2000). Control of work pacing (four questions) and social climate (three questions) were assessed on a 5-point scale from 1 ‘very seldom or never’ to 5 ‘very often or always’ (Dallner et al. 2000). Self-reported duration of working with hands above shoulder height was rated on a 6-point scale: 0 ‘Never’, 1 ‘Very small part of the time’, 2 ‘Approximately 25% of the time’, 3 ‘Approximately 50% of the time’, 4 ‘Approximately 75% of the time’, and 5 ‘Almost all the time’.

### Data analyses

Descriptive statistics were calculated using SPSS (IBM25.0). Compositional data analyses were conducted in R (version 3.5.1) (RStudio, Boston, MA, USA) using the ‘lme4’, ‘lmerTest’, ‘compositions’, and ‘robCompositions’ packages. *p* < 0.05 was considered to show statistical significance.

Percentages of time in categories of arm elevation, trapezius activity, and neck/shoulder load were summarised in standard cumulative distributions as well as in in terms of geometric means (Pedisic et al. 2017). These geometric means and other descriptive statistics were reported for the total sample as well as for the construction and healthcare sectors separately. However, data for the two sectors were merged when determining the associations between exposures and NSPi to achieve a sufficient sample size, and the largest possible contrast in exposure between workers.

Following CoDA procedures, each exposure composition of the total sample was then expressed as a set of isometric log-ratio (ilr)-coordinates (Dumuid et al. 2018; Gupta et al. 2020). The number of ilr-coordinates for an *n*-part composition is *n*-1. Thus, the compositions of arm elevation and trapezius activity (both 3-part compositions) were transformed into sets of two ilr-coordinates, while neck/shoulder load (a 4-part composition) was expressed in terms of three ilr-coordinates. Equations defining the ilr-coordinates are presented in Appendix A. After ilr transformation, data were analysed using standard statistical methods (Chastin et al. 2015; Dumuid et al. 2018).

NSPi was analysed under the assumption that ratings could be treated as values on an equidistant scale from 0 to 3. Associations between the course of NSPi during the 2-year follow-up and the compositions of arm elevation, trapezius activity, and neck/shoulder load were analysed using three separate linear mixed models with random intercept for subject. Each model included the ilr-coordinates, time during follow-up (as a continuous variable from 0 to 4 with the value ‘0’ corresponding to baseline), and interactions between the ilr-coordinates and time. Visually inspected quantile–quantile plots indicated that normality assumptions of residuals after regression were met. To test whether each complete composition was associated with the course of NSPi, the model with only time as independent variable was compared with that including ilr-coordinates, time and interactions between the coordinates and time, using a likelihood ratio test.

For each exposure (arm elevation, trapezius activity, neck/shoulder load), a set of regression models (cf. Appendix B) was constructed so that each compositional part (cf. Appendix A) was represented as the primary independent variable, with the remaining ilr-coordinates included as confounders. This “rotation” of variables allows for determination of the effect of each part, relative to all others in the composition. Thus, the main effect (β1, cf. Appendix B) indicated the relative contribution of the exposure of interest to NSPi at baseline. The interaction effect (β4 for arm elevation and trapezius activity; β5 for shoulder load, cf. Appendix B) indicated the relative contribution of the exposure level of interest to the change in NSPi over the 2-year follow-up, beyond the effect of time alone. The effect of time is accounted for by β3 for arm elevation and trapezius activity, and by β4 for shoulder load.

Candidate confounders were occupational sector, gender, seniority, BMI, social climate, control of work pacing, and pain in the neck and shoulder region during the 12 months preceding baseline. Whether to include these candidates in adjusted models was assessed in two steps, both of which were required for inclusion. (1) A statistically significant association between the candidate confounder and NSPi (either as a main effect or as an interaction with time); (2) an at least 10% change in the main exposure effect when adding the potential confounder to the crude model, or a 10% change in the interaction exposure*time when adding the interaction confounder*time. All accepted confounding variables for a particular exposure were then added into an adjusted model, which was otherwise constructed as described above.

Results of the adjusted regression analyses were illustrated using isotemporal substitution, in which time was added and subtracted from the geometric mean of each exposure composition in 30 min increments according to the one-to-all substitution method (Dumuid et al. 2018). The substitutions of time were all within the 95% range of exposures observed in the source data.

#### Sensitivity analysis

Separate models were resolved for workers with and without pain at baseline, to assess whether the association between the course of NSPi and arm elevation, trapezius activity, and neck/shoulder load differed for these two groups.

### Stability of exposures

An indication of whether exposures were stable throughout the 2-year follow-up was obtained by inspecting each individual’s repeated self-reports for any obvious systematic change over time.

## Results

**Fig. 1:**
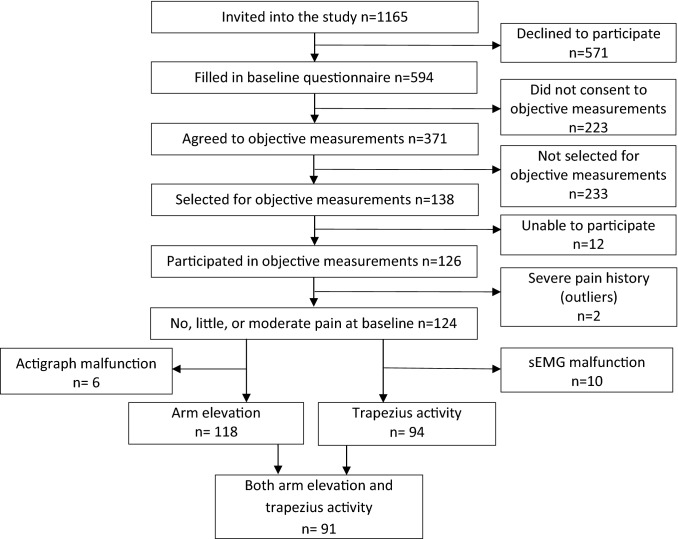
Flow chart for workers included in the objective measurements

Among the 138 selected workers, 12 were unable to participate due to various practical reasons (Fig. 1). Two workers had severe pain in both the neck and dominant shoulder at baseline, and reported to have pain daily during the 12 months preceding baseline. These two workers were considered to be outliers and were excluded from the dataset. Among the remaining 124 workers, either accelerometry or sEMG data were available from 121 workers (construction *n* = 60; healthcare *n* = 61). At baseline, 97% of the workers answered the questions on NSPi; after 24 months, 52% answered these questions.

### Sample characteristics

Almost all construction workers were male; among the healthcare workers, about a quarter were male (Table 1). Participants were on average 42.0 years, they had 16.5 years of work experience, and worked 36.7 h per week. Half of the workers perceived themselves to be in good to excellent health. Two-thirds of the workers reported little or moderate NSPi at baseline; less than half reported pain longer than 30 days in the year prior to baseline.

**Table 1:** Participant characteristics at baseline

	Total *n* = 121				Construction *n* = 60				Healthcare *n* = 61			
	*n*	(%)	Mean	(SD)	*n*	(%)	Mean	(SD)	*n*	(%)	Mean	(SD)
Age (years)			42.0	(12.0)			39.6	(13.4)			44.4	(10.0)
Gender												
Male	73	(60)			59	(98)			14	(23)		
Female	48	(40)			1	(2)			47	(77)		
BMI												
Normal (< 25 kg/m^2^)	60	(50)			25	(42)			35	(57)		
Overweight (≥ 25 kg/m^2^)	61	(50)			35	(58)			26	(43)		
General health												
Poor-fair	17	(14)			8	(13)			9	(15)		
Good	46	(38)			24	(40)			22	(36)		
Very good—excellent	58	(48)			28	(47)			30	(49)		
Seniority (years)			16.5	(11.3)			17.4	(12.7)			15.7	(9.8)
Work hours (per week)			36.7	(4.3)			37.8	(4.1)			35.7	(4.3)
Work pacing (1–5)			2.9	(0.8)			3.0	(0.8)			2.8	(0.8)
Social climate (1–5)			3.2	(0.6)			3.1	(0.5)			3.2	(0.6)
Shoulder strength (N)			219.7	(90.3)			276.6	(79.5)			163.8	(63.8)
NSPi at baseline												
No pain	45	(38)			29	(50)			16	(27)		
A little pain	35	(30)			18	(31)			17	(29)		
Moderate pain	37	(32)			11	(19)			26	(44)		
NSP 12 months preceding baseline												
0 days	42	(34)			28	(47)			14	(23)		
1–7 days	12	(10)			4	(7)			8	(13)		
8–30 days	17	(14)			5	(8)			12	(20)		
> 30 days, not daily	36	(30)			18	(30)			18	(29)		
Daily	14	(12)			5	(8)			9	(15)		

### Exposure compositions

**Fig. 2:**
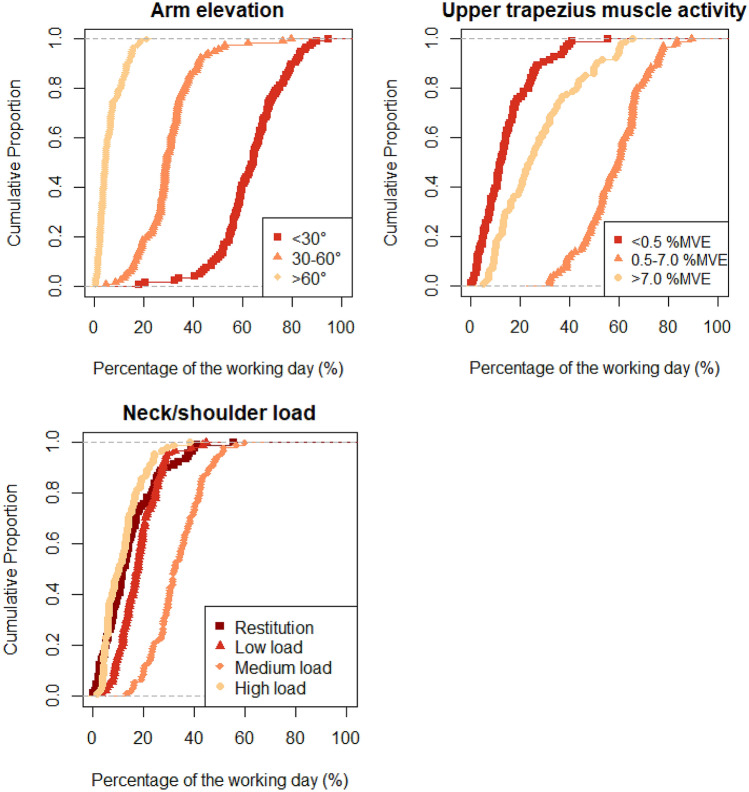
Cumulative probability distributions for arm elevation, upper trapezius muscle activity, and neck/shoulder load

The compositional means (Table 2) and the cumulative distributions (Fig. 2) show that the majority of the working time was spent with the arm elevated < 30°, with trapezius activity between 0.5 and 7.0% MVE, and with low neck/shoulder load. Some differences existed in exposure composition between the sectors, the largest of which were for duration arm elevation < 30° and low load: on average, healthcare workers spent 10% (45 min) and 9% (41 min) more time, respectively, in these exposure levels than construction workers.

**Table 2:** Geometric means of arm elevation (*n* = 118), trapezius activity (*n* = 94), and neck/shoulder load (*n* = 91)

Arm elevation	Geometric mean of the proportion of the working day (%time)		
	Total sample (*n* = 118)	Construction (*n* = 59)	Healthcare (*n* = 59)
< 30°	65	60	70
30–60°	30	33	26
> 60°	5	7	3

Trapezius activity	Total sample (*n* = 94)	Construction (*n* = 39)	Healthcare (*n* = 55)
< 0.5%MVE	12	15	10
0.5–7.0%MVE	63	61	64
> 7.0%MVE	25	24	26

Neck/shoulder load	Total sample	Construction (*n* = 38)	Healthcare (*n* = 53)
Restitution	12	15	10
Low load	42	37	46
Medium load	35	36	34
High load	11	12	10

Fifty-four percent of the workers reported changes in working with hands above shoulder height during the 2-year follow-up by at least one category (a change in 25% duration or almost 2 h during a working day). These workers reported either an increase, a decrease, or varying exposure during follow-up.

### Estimated average pain during follow-up

Pain at baseline was estimated by the adjusted model to be 0.9 on average (on a scale from 0–3), and to increase by between 0.01 and 0.02 every 6 months during the course of the 2-year follow-up (Table 3, next to last column; Figs. C1–C3 in Appendix C, the solid line in all panels).

**Table 3:** Crude and adjusted models describing the association of arm elevation (*n* = 118), trapezius activity (*n* = 94), and neck/shoulder load (*n* = 91) with NSPi, and the isotemporal substitutions estimating the change in NSPi when adding 30 min of exposure in the category at hand

Arm elevation (*n* = 118)	Crude model			Adjusted model^a^			Isotemporal substitution estimates	
	β	SE	*p* value	Β	SE	*p* value	Mean time course	+ 30 min
Intercept	0.57	0.23	**0.014**	0.18	0.20	0.361	0.91	
Time	0.11	0.06	0.085	0.10	0.06	0.100	0.01	
< 30° (vs > 30°)	**0.37**	**0.15**	**0.015**	0.20	0.13	0.126		0.05
< 30° (vs > 30°)*time	− 0.07	0.04	0.089	− 0.06	0.04	0.097		− 0.01
30–60° (vs < 30° and > 60°)	− 0.31	0.20	0.120	− 0.22	0.16	0.161		− 0.05
30–60° (vs < 30° and > 60°)*Time	0.03	0.05	0.539	0.03	0.05	0.534		0.02
> 60° (vs < 60°)	− 0.07	0.13	0.610	0.03	0.11	0.820		0.03
> 60° (vs < 60°)*time	− 0.06	0.05	0.243	0.03	0.03	0.324		0.03

Trapezius activity (*n* = 94)	Crude model			Adjusted model^b^			Mean time course	+ 30 min
	β	SE	*p* value	β	SE	*p* value		
Intercept	0.53	0.25	**0.034**	0.03	0.41	0.941	0.90	
Time	**0.14**	**0.07**	**0.045**	**0.43**	**0.16**	**0.006**	**0.02**	
< 0.5%MVE (vs > 0.5%MVE)	− **0.26**	**0.13**	**0.041**	− **0.21**	**0.10**	**0.045**		− **0.09**
< 0.5%MVE (vs > 0.5%MVE)*time	0.03	0.04	0.327	0.05	0.03	0.113		0.04
0.5–7.0%MVE (vs < 0.5% & > 7.0%MVE)	0.30	0.22	0.173	0.32	0.18	0.072		0.08
0.5–7.0%MVE (vs < 0.5% & > 7.0%MVE)*Time	− **0.13**	**0.06**	**0.040**	− **0.13**	**0.06**	**0.037**		− **0.01**
> 7.0%MVE (vs < 7.0%MVE)	− 0.04	0.14	0.774	− 0.11	0.11	0.330		− 0.03
> 7.0%MVE (vs < 7.0%MVE)*time	**0.09**	**0.04**	**0.019**	0.07	0.04	0.067		0.04

Neck/Shoulder(*n* = 91)	Crude model			Adjusted model^b^			Mean time course	+ 30 min
	β	SE	*p* value	β	SE	*p* value		
Intercept	0.48	0.25	0.057	0.09	0.40	0.819	0.89	
Time	**0.17**	**0.07**	**0.022**	**0.40**	**0.16**	**0.010**	**0.02**	
Restitution (*vs* shoulder load)	− **0.28**	**0.10**	**0.008**	− 0.17	0.09	0.053		−0.08
Restitution (*vs* shoulder load)*Time	0.02	0.03	0.498	0.03	0.03	0.223		0.04
Low load (vs restitution, medium, high load)	**0.48**	**0.21**	**0.026**	**0.40**	**0.18**	0.027		**0.09**
Low load (vs restitution, medium, high load)*Time	− 0.07	0.06	0.260	− 0.09	0.06	0.132		0.00
Medium load (vs restitution, low, high load)	− 0.12	0.31	0.697	− 0.17	0.25	0.510		− 0.04
*Medium* load (vs restitution, low, high load)*Time	− 0.07	0.10	0.445	−0.02	0.09	0.874		0.02
High load (vs restitution, low, medium load)	− 0.09	0.20	0.661	− 0.06	0.16	0.536		− 0.03
High load (vs restitution, low, medium load)*Time	**0.12**	**0.06**	**0.047**	0.07	0.06	0.276		0.05

Bold face mark associations with *p* < 0.05

^a^Adjusted for gender, sector, and NSP duration in the 12 months preceding baseline

^b^Adjusted for gender, sector, NSP duration in the 12 months preceding baseline, social climate, social climate*time, control of work pacing, control of work pacing*time

^c^Adjusted for sector and NSP duration in the 12 months preceding baseline, social climate, social climate*time, control of work pacing, control of work pacing*time

### Arm elevation

The overall composition of arm elevation was weakly associated with the development of NSPi over the 2-year follow-up (likelihood ratio test *p* = 0.108). None of the individual exposure levels of arm elevation showed a statistically significant association with the course of NSPi over the 2-year follow-up in the adjusted models, and all associations were small in size (Table 3, Fig. C1). The strongest associations for baseline NSPi were found for time spent < 30° and in 30–60°: reallocating 30 min into these exposure intervals (at the expense of time in the other exposure intervals) was associated with a 0.05 lower and a 0.05 higher NPSi at baseline, respectively. The most certain influence on the change in NSPi over the course of the 2-year follow-up occurred for time spent < 30°: 30 min more time spent in that exposure interval was associated with a reduction of 0.01 in NSPi every 6 months, as compared to the group’s average increase of 0.01.

### Upper trapezius muscle activity

The overall composition of trapezius activity showed a statistically significant association with the development of NSPi over the 2-year follow-up (likelihood ratio test *p* = 0.029).

Regarding associations between trapezius activity categories and NSPi at baseline, the largest and most certain were found for time spent < 0.5% MVE (*p* = 0.045) and in 0.5–7.0% MVE (*p* = 0.072). Thirty more minutes spent in < 0.5% MVE than the geometric mean (53 min) at the cost of the other categories, was associated with less pain at baseline, by 0.09, while 30 min more in 0.5–7.0% MVE was associated with 0.08 more pain (Table 3; Fig. C2).

During the 2-year follow-up, more time spent in < 0.5%MVE (*p* = 0.113) and > 7.0%MVE (*p* = 0.067) were associated with an increase in pain. For both categories, 30 min more time was associated with a 0.04 increase in NSPi every 6 months, which is 0.02 higher than the average increase in NSPi (Table 3; Fig. C2). In contrast, more time spent in 0.5–7.0%MVE was associated with a reduced NSPi (*p* = 0.037); 30 min more time spent at this exposure level was associated with a 0.01 decline in pain every 6 months, which is 0.03 lower than the average increase of 0.02 NSPi (Table 3; Fig. C2).

### Neck/shoulder load

The association between the NSPi over the 2-year follow-up and the entire composition of neck/shoulder load was statistically significant (likelihood ratio test *p* = 0.022).

The individual contributions of each category of neck/shoulder load to the course of NSPi during the 2-year follow-up were small; only the effects on NSPi at baseline of low load and of restitution were reasonably certain (*p* = 0.027 and *p* = 0.053, respectively; Table 3). These two exposure categories also contributed the most to explaining NSPi at baseline: increasing time by 30 min relative to their respective geometric means (in exchange for 30 min in the other categories) were associated with a 0.08 lower NSPi at baseline for restitution and an increase in 0.09 for low load (Table 3). High load had the largest influence on the change in pain over the 2-year follow-up: 30 min more of high load than the geometric mean (at the expense of all lower loads) was associated with an increase in NSPi of 0.05 every 6 months, i.e. 0.03 higher than the average of 0.02 NSPi (Table 3; Fig. C3).

### Sensitivity analyses

Associations between time spent in arm elevation and the course of NSPi were small and not statistically significant for workers regardless of pain status at baseline (Tables D1 and D2).

Associations between trapezius activity and changes in NSPi during the 2-year follow-up were larger for those with pain at baseline (Tables D2) than for those without pain (Tables D2).

The associations of restitution, medium, and high load with changes in NSPi over the 2-year follow-up had the same direction for workers who were pain-free at baseline as for the whole sample, but the effect sizes for medium and high load were considerably larger, and now statistically significant (Tables D1). Workers with and without pain at baseline showed opposite associations between neck/shoulder load categories and changes in NSPi over time. When associations were negative for workers with pain at baseline (Tables D2), they were positive for those without pain (Tables D1); and vice versa.

## Discussion

Among construction and healthcare workers, the compositions of arm elevation, trapezius activity, neck/shoulder load, were weakly associated with the course of NSPi over a 2-year follow-up, and associations were even uncertain. In terms of effect sizes, the combined exposure metric, i.e. neck/shoulder load, was no better in explaining the course of NSPi than each of the compositions of arm elevation or trapezius muscle activity separately. However, among workers who were pain-free at baseline, we found indications that neck/shoulder load may explain the course of NSPi better than either arm elevation or trapezius activity separately.

Previous studies have suggested that composite exposures are more strongly related to musculoskeletal pain than single exposures, but our study does not support this conviction (Andersen et al. 2003; Jakobsen et al. 2018; van der Molen et al. 2017). First, even though the posture and activity categories we chose, and consequently the combinations in our composite metric, are in line with those typically found in the literature, the exposure categories for our composite metric may have needed more contrast to provide stronger associations with NSPi. For example, increases in muscle activity are more pronounced beyond 60° arm elevation, rather than the 30° used as a discrimination level in the present study (Brookham et al. 2010). Other categorizations may have led to different associations with NSPi, and the sensitivity of our outcome to the threshold limit selected for, e.g. the highest-exposure category, is an interesting issue for further studies.

Second, we used objective exposure assessments, while previous studies have mainly been based on exposures assessed by self-reports or experts; this often leads to more consistent associations with pain than objective assessments, likely related to common methods bias, attribution, and preconception (Andersen et al. 2003; Bodin et al. 2012; Coenen et al. 2016; Jakobsen et al. 2018; Koch et al. 2017; Svendsen et al. 2013). On the other hand, these studies may have combined several different exposures, including repetitiveness of arm movements, force use, duration of neck flexion, and lack of recovery, and thus encompass a more comprehensive composite metric of neck/shoulder load than that reflected by the two exposures used in our study (Andersen et al. 2003; Jakobsen et al. 2018; Svendsen et al. 2013). Third, it is possible that a neck/shoulder metric would be more strongly associated with the onset of pain, as indicated by the more pronounced association in our own and other pain-free populations at baseline, than in a group of workers among whom several already have pain at baseline (Andersen et al. 2003; Feveile et al. 2002). An optimal neck/shoulder metric using objective measures remains to be developed, and its benefits beyond separate assessments remains to be documented in future research.

The associations between exposure and pain found in our study were weak and uncertain for all three objectively assessed exposures. Although physical workloads in our sample of construction and healthcare workers were comparable to that in other occupations considered to be physically demanding (Nordander et al. 2016), the exposures may not have been sufficiently extreme to lead to pronounced effects on pain. On average, only 1% (5 min) of the working day was spent with arms elevated > 90°, and time spent > 60° was less than 10% (45 min) for most workers in our sample. Similarly, the occurrence of ‘high muscle activity’ (i.e. > 7.0% MVE) and ‘high neck/shoulder load’ (i.e. arm elevation > 30° with trapezius activity > 7.0% MVE) may have been too little to have detectable consequences for NSPi within the 2-year period covered by the study. To this end, a longer follow-up may be needed to detect associations; or other exposure variables, such as exposure bout length, exposure variation, peak exposures, or movement velocity might be more strongly associated with NSPi than the overall exposure duration (Balogh et al. 2019; Coenen et al. 2016; Mathiassen 2006; Nordander et al. 2016). The relatively small sample allowed us logistically to measure several exposures simultaneously, but the notable loss of data, particularly for trapezius activity, prevented us from analysing the data separately for each sector. While merging the sectors is an advantage in terms of sample size and exposure contrasts, it will conceal possible differences in associations between the sectors.

Furthermore, although objectively assessed biomechanical exposures are more accurate than subjectively assessed exposures, we acknowledge some limitations related to our assessment of the exposures. We measured exposures for only one day at baseline, and used the result as an estimate of long-term individual averages. These individual exposure estimates were likely somewhat uncertain, since exposure may vary between days at work (Heiden et al. 2019; Wahlstrom et al. 2016, 2010). Uncertain exposures are known to attenuate exposure–outcome associations in studies applying an individual-based strategy (Nordander et al. 2004; Tielemans et al. 1998), and thus ‘true’ associations between exposure and NSPi may have been stronger than those determined in the present study (Hansson et al. 2010; Nordander et al. 2004). Also, systematic changes in exposures may have occurred within individuals over the 2-year follow-up. Inspection of the repeated self-reported arm elevation suggested that changes in exposure may have occurred for a part of the sample, so some influence on associations—either over- or under-estimation—cannot be ruled out.

Additionally, the results need to be interpreted with caution, because those consenting to participate, particularly in the technical measurement group, may have been a selected group of workers. The prevalence of self-reported neck/shoulder pain among our participants (62%) was larger than found in a representative sample of Norwegian construction and healthcare personnel (31–52%) (NOA 2016). Finally, a healthy worker survivor effect, as indicated by a relatively good self-perceived general health, may have attenuated any ‘true’ associations between exposure and pain.

### Future studies

We recommend that future studies based on exposures or outcomes expressed in terms of time-use should consider using compositional data analysis to obtain valid associations between exposures and outcomes (Pedisic et al. 2017). Future studies should investigate the ability to predict NSP with composite metrics using other thresholds for arm elevation and trapezius muscle activity than those adopted by us, and may even investigate the effect of adding even more exposures to a combined metric. Furthermore, next to repeated outcome data, future studies should also collect repeated exposure data so as to reduce exposure uncertainty at the individual level and to keep track of possible systematic changes over time.

## Conclusion

The compositional exposure metric ‘neck/shoulder load’, which was a composite metric of arm elevation or trapezius muscle activity, was not associated any stronger with the course of NSPi among construction and healthcare workers than each of its constituent parts alone. However, our findings suggest that neck/shoulder load may be more strongly related to NSPi among workers who were pain-free at baseline than among workers with pain at entry.

## Electronic supplementary material

Below is the link to the electronic supplementary material.

Supplementary file1 (DOCX 16 KB)

Supplementary file2 (DOCX 17 KB)

Supplementary file3 (DOCX 512 KB)

Supplementary file4 (DOCX 26 KB)

## Data Availability

Data are available upon request.

## Code availability

Software applications and custom code used for this manuscript are available.
