# Supplementary material for: Can a metric combining arm elevation and trapezius muscle activity predict neck/shoulder pain? A prospective cohort study in construction and healthcare
Source: Int Arch Occup Environ Health. 2020 Dec 5;94(4):647–58. doi: 10.1007/s00420-020-01610-w (PMC8068682; doi:10.1007/s00420-020-01610-w)
Supplement: Supplementary file 1 — Supplementary file1 (DOCX 16 KB) [file 420_2020_1610_MOESM1_ESM.docx]

# **Can a metric combining arm elevation and trapezius muscle activity predict neck/shoulder pain? A prospective cohort study in construction and healthcare**

Suzanne Lerato Merkus^1^ (ORCID 0000-0003-0945-3738)

Svend Erik Mathiassen (ORCID 0000-0003-1443-6211)^2^

Lars-Kristian Lunde (ORCID 0000-0001-6219-9244) ^1^

Markus Koch (PhD) ^1^

Morten Wærsted (ORCID 0000-0002-9570-2181) ^1^

Mikael Forsman (ORCID 0000-0001-5777-4232)^3,4^

Stein Knardahl (ORCID 0000-0002-7300-8519) ^1^

Kaj Bo Veiersted (ORCID 0000-0003-1221-384X)^1^

^1^ National Institute of Occupational Health, Oslo, Norway

^2^ Centre for Musculoskeletal Research, Department of Occupational and Public Health Sciences, University of Gävle, Gävle, Sweden

^3^ School of Engineering Sciences in Chemistry, Biotechnology and Health, KTH Royal Institute of Technology, Huddinge, Sweden

^4^ IMM Institute of Environmental Medicine, Karolinska Institutet, Stockholm, Sweden

**Corresponding author:** Suzanne L. Merkus**,** National Institute of Occupational Health, Pb 5330 Majorstuen, 0304 Oslo, Norway**.** E-mail: [suzanne.merkus@stami.no](mailto:suzanne.merkus@stami.no). Phone: (+47) 2319 5100. [www.stami.no](http://www.stami.no)

## Journal

International Archives of Occupational and Environmental Health

## Appendix A - isometric log-ratio transformations

### Arm elevation time-use composition

The ilr-coordinates for the contribution of duration <30° relative to durations 30-60° and >60° were computed as follows:

$${ilr}_{1}=\sqrt{\frac{2}{3}}\ln\left( \frac{<30^{\circ}}{\sqrt[2]{30-60^{\circ}* >60^{\circ}}} \right)$$

$${ilr}_{2}=\sqrt{\frac{1}{2}}\ln\left( \frac{30-60^{\circ}}{>60^{\circ}} \right)$$

The ilr-coordinates for the contribution of duration 30-60° relative to durations <30° and >60° were computed as follows:

$${ilr}_{1}=\sqrt{\frac{2}{3}}\ln\left( \frac{30-60^{\circ}}{\sqrt[2]{<30^{\circ}* >60^{\circ}}} \right)$$

$${ilr}_{2}=\sqrt{\frac{1}{2}}\ln\left( \frac{<30^{\circ}}{>60^{\circ}} \right)$$

The ilr-coordinates for the contribution of duration >60° relative to durations <30° and 30-60° were computed as follows:

$${ilr}_{1}=\sqrt{\frac{2}{3}}\ln\left( \frac{>60^{\circ}}{\sqrt[2]{<30^{\circ}*30-60^{\circ}}} \right)$$

$${ilr}_{2}=\sqrt{\frac{1}{2}}\ln\left( \frac{<30^{\circ}}{30-60^{\circ}} \right)$$

### Upper trapezius muscle activity time-use composition

The ilr-coordinates for the contribution of duration <0.5 %MVE relative to durations 0.5-7.0 %MVE and >7.0 %MVE were computed as follows:

$${ilr}_{1}=\sqrt{\frac{2}{3}}\ln\left( \frac{<0.5 \%MVE}{\sqrt[2]{0.5-7.0 \%MVE* >7.0 \%MVE}} \right)$$

$${ilr}_{2}=\sqrt{\frac{1}{2}}\ln\left( \frac{0.5-7.0 \%MVE}{>7.0 \%MVE} \right)$$

The ilr-coordinates for the contribution of duration 0.5-7.0 %MVE relative to durations <0.5 %MVE and >7.0 %MVE were computed as follows:

$${ilr}_{1}=\sqrt{\frac{2}{3}}\ln\left( \frac{0.5-7.0 \%MVE}{\sqrt[2]{<0.5 \%MVE* >7.0 \%MVE}} \right)$$

$${ilr}_{2}=\sqrt{\frac{1}{2}}\ln\left( \frac{<0.5 \%MVE}{>7.0 \%MVE} \right)$$

The ilr-coordinates for the contribution of duration >7.0 %MVE relative to durations <0.5 %MVE and 0.5-7.0 %MVE were computed as follows:

$${ilr}_{1}=\sqrt{\frac{2}{3}}\ln\left( \frac{>7.0 \%MVE}{\sqrt[2]{<0.5 \%MVE*0.5-7.0 \%MVE}} \right)$$

$${ilr}_{2}=\sqrt{\frac{1}{2}}\ln\left( \frac{<0.5 \%MVE}{0.5-7.0 \%MVE} \right)$$

### Neck/shoulder load time-use composition

The ilr-coordinates for the contribution of duration in restitution relative to durations in low, medium, and high load were computed as follows:

$${ilr}_{1}=\sqrt{\frac{3}{4}}\ln\left( \frac{restitution}{\sqrt[3]{low load*medium load*high load}} \right)$$

$${ilr}_{2}=\sqrt{\frac{2}{3}}\ln\left( \frac{low load}{\sqrt[2]{medium load*high load}} \right)$$

$${ilr}_{3}=\sqrt{\frac{1}{2}}\ln\left( \frac{medium load}{high load} \right)$$

The ilr-coordinates for the contribution of duration in low load relative to durations in restitution, medium, and high load were computed as follows:

$${ilr}_{1}=\sqrt{\frac{3}{4}}\ln\left( \frac{low load}{\sqrt[3]{restitution*medium load*high load}} \right)$$

$${ilr}_{2}=\sqrt{\frac{2}{3}}\ln\left( \frac{restitution}{\sqrt[2]{medium load*high load}} \right)$$

$${ilr}_{3}=\sqrt{\frac{1}{2}}\ln\left( \frac{medium load}{high load} \right)$$

The ilr-coordinates for the contribution of duration in medium load relative to durations in restitution, low, and high load were computed as follows:

$${ilr}_{1}=\sqrt{\frac{3}{4}}\ln\left( \frac{medium load}{\sqrt[3]{restitution*low load*high load}} \right)$$

$${ilr}_{2}=\sqrt{\frac{2}{3}}\ln\left( \frac{restitution}{\sqrt[2]{low load*high load}} \right)$$

$${ilr}_{3}=\sqrt{\frac{1}{2}}\ln\left( \frac{low load}{high load} \right)$$

The ilr-coordinates for the contribution of duration in high load relative to durations in restitution, low, and medium load were computed as follows:

$${ilr}_{1}=\sqrt{\frac{3}{4}}\ln\left( \frac{high load}{\sqrt[3]{restitution*low load*medium load}} \right)$$

$${ilr}_{2}=\sqrt{\frac{2}{3}}\ln\left( \frac{restitution}{\sqrt[2]{low load*medium load}} \right)$$

$${ilr}_{3}=\sqrt{\frac{1}{2}}\ln\left( \frac{low load}{medium load} \right)$$
