# Supplementary material for: Can a metric combining arm elevation and trapezius muscle activity predict neck/shoulder pain? A prospective cohort study in construction and healthcare
Source: Int Arch Occup Environ Health. 2020 Dec 5;94(4):647–58. doi: 10.1007/s00420-020-01610-w (PMC8068682; doi:10.1007/s00420-020-01610-w)
Supplement: Supplementary file 2 — Supplementary file2 (DOCX 17 KB) [file 420_2020_1610_MOESM2_ESM.docx]

# **Can a metric combining arm elevation and trapezius muscle activity predict neck/shoulder pain? A prospective cohort study in construction and healthcare**

Suzanne Lerato Merkus^1^ (ORCID 0000-0003-0945-3738)

Svend Erik Mathiassen (ORCID 0000-0003-1443-6211)^2^

Lars-Kristian Lunde (ORCID 0000-0001-6219-9244) ^1^

Markus Koch (PhD) ^1^

Morten Wærsted (ORCID 0000-0002-9570-2181) ^1^

Mikael Forsman (ORCID 0000-0001-5777-4232)^3,4^

Stein Knardahl (ORCID 0000-0002-7300-8519) ^1^

Kaj Bo Veiersted (ORCID 0000-0003-1221-384X)^1^

^1^ National Institute of Occupational Health, Oslo, Norway

^2^ Centre for Musculoskeletal Research, Department of Occupational and Public Health Sciences, University of Gävle, Gävle, Sweden

^3^ School of Engineering Sciences in Chemistry, Biotechnology and Health, KTH Royal Institute of Technology, Huddinge, Sweden

^4^ IMM Institute of Environmental Medicine, Karolinska Institutet, Stockholm, Sweden

**Corresponding author:** Suzanne L. Merkus**,** National Institute of Occupational Health, Pb 5330 Majorstuen, 0304 Oslo, Norway**.** E-mail: [suzanne.merkus@stami.no](mailto:suzanne.merkus@stami.no). Phone: (+47) 2319 5100. [www.stami.no](http://www.stami.no)

## Journal

International Archives of Occupational and Environmental Health

## Appendix B. Mixed models regression equations

### Upper arm elevation and upper trapezius muscle activity

The generic compositional mixed models regression equations for arm elevation and upper trapezius muscle activity were defined as:

${NSPi}_{jt}= \beta^{0}+ \beta^{1}*{ilr.1}_{j0}+\beta^{2}*{ilr.2}_{j0} + \beta^{3}*time+\beta^{4}*{ilr.1}_{j0}*time+ \beta^{5}*{ilr.2}_{j0}*time+\sum_{k=6}^{K+5} \beta^{k}*{Z^{k}}_{jt}+\sum_{m=K+6}^{M+K+5} \beta^{m}*{G^{m}}_{j}+u_{j}+ \epsilon_{jt}$

where ${NSPi}_{jt}$ is neck and shoulder pain intensity for subject *j* at time *t,* $\beta^{0}$ is the intercept, ${ilr.1}_{j0}$ is the first ilr-coordinate for subject *j* at time *0*, and $\beta^{1}$ is the regression coefficient for the first ilr-coordinate; ${ilr.2}_{j0}$ is the second ilr-coordinate for subject *j* at time *0*, and $\beta^{2}$ is the regression coefficient for the second ilr-coordinate; *t* is time, and $\beta^{3}$ is the regression coefficient for time;${ilr.1}_{j0}*time$ is the interaction term between the first ilr-coordinate for subject *j* and time, and $\beta^{4}$ is the regression coefficient for subject *j* for this interaction term; ${ilr.2}_{j0}*time$ is the interaction term between the second ilr-coordinate for subject *j* and time, and $\beta^{5}$ is the regression coefficient for subject *j* for this interaction term. Thereafter follow general forms for the correction of time-dependent and time-independent confounders. ${Z^{k}}_{jt}$ is time-dependent confounder *k* for subject *j* at time *t*, and $\beta^{k}$ is the regression coefficient for the time dependent confounder, *K* is the number of time-dependent confounders; ${G^{m}}_{j}$ is the time-independent confounder *m* for subject *j,*  and $\beta^{m}$ is the regression coefficient for the time-independent confounder, *M* is the number of time-independent confounders. Lastly, $u_{j}$ is the random intercept and $\epsilon_{jt}$ is the ‘error’ for subject *j* at time *t.*

### Neck/shoulder load

The generic compositional mixed models regression equation for neck/shoulder load was defined as:

${NSPi}_{jt}= \beta^{0}+ \beta^{1}*{ilr.1}_{j0}+\beta^{2}*{ilr.2}_{j0} + \beta^{3}*{ilr.3}_{j0} + \beta^{4}*time+\beta^{5}*{ilr.1}_{j0}*time+ \beta^{6}*{ilr.2}_{j0}*time+\beta^{7}*{ilr.3}_{j0}*time+ \sum_{k=8}^{K+7} \beta^{k}*{Z^{k}}_{jt}+\sum_{m=K+8}^{M+K+7} \beta^{m}*{G^{m}}_{j}+u_{j}+ \epsilon_{jt}$

where ${NSPi}_{jt}$ is neck and shoulder pain intensity for subject *j* at time *t,* $\beta^{0}$ is the intercept, ${ilr.1}_{j0}$ is the first ilr-coordinate for subject *j* at time *0*, and $\beta^{1}$ is the regression coefficient for the first ilr-coordinate; ${ilr.2}_{j0}$ is the second ilr-coordinate for subject *j* at time *0*, and $\beta^{2}$ is the regression coefficient for the second ilr-coordinate; ${ilr.3}_{j0}$ is the third ilr-coordinate for subject *j* at time *0*, and $\beta^{3}$ is the regression coefficient for the third ilr-coordinate; *t* is time, and $\beta^{4}$ is the regression coefficient for time;${ilr.1}_{j0}*time$ is the interaction term between the first ilr-coordinate for subject *j* and time, and $\beta^{5}$ is the regression coefficient for subject *j* for this interaction term; ${ilr.2}_{j0}*time$ is the interaction term between the second ilr-coordinate for subject *j* and time, and $\beta^{6}$ is the regression coefficient for subject *j* for this interaction term; ${ilr.3}_{j0}*time$ is the interaction term between the third ilr-coordinate for subject *j* and time, and $\beta^{7}$ is the regression coefficient for subject *j* for this interaction term. Thereafter follow general forms for the correction of time-dependent and time-independent confounders. ${Z^{k}}_{jt}$ is time-dependent confounder *k* for subject *j* at time *t*, and $\beta^{k}$ is the regression coefficient for the time dependent confounder, *K* is the number of time-dependent confounders; ${G^{m}}_{j}$ is the time-independent confounder *m* for subject *j,*  and $\beta^{m}$ is the regression coefficient for the time-independent confounder, *M* is the number of time-independent confounders. Lastly, $u_{j}$ is the random intercept and $\epsilon_{jt}$ is the ‘error’ for subject *j* at time *t.*
