# Supplementary material for: Can a metric combining arm elevation and trapezius muscle activity predict neck/shoulder pain? A prospective cohort study in construction and healthcare
Source: Int Arch Occup Environ Health. 2020 Dec 5;94(4):647–58. doi: 10.1007/s00420-020-01610-w (PMC8068682; doi:10.1007/s00420-020-01610-w)
Supplement: Supplementary file 3 — Supplementary file3 (DOCX 512 KB) [file 420_2020_1610_MOESM3_ESM.docx]

# **Can a metric combining arm elevation and trapezius muscle activity predict neck/shoulder pain? A prospective cohort study in construction and healthcare**

Suzanne Lerato Merkus^1^ (ORCID 0000-0003-0945-3738)

Svend Erik Mathiassen (ORCID 0000-0003-1443-6211)^2^

Lars-Kristian Lunde (ORCID 0000-0001-6219-9244) ^1^

Markus Koch (PhD) ^1^

Morten Wærsted (ORCID 0000-0002-9570-2181) ^1^

Mikael Forsman (ORCID 0000-0001-5777-4232)^3,4^

Stein Knardahl (ORCID 0000-0002-7300-8519) ^1^

Kaj Bo Veiersted (ORCID 0000-0003-1221-384X)^1^

^1^ National Institute of Occupational Health, Oslo, Norway

^2^ Centre for Musculoskeletal Research, Department of Occupational and Public Health Sciences, University of Gävle, Gävle, Sweden

^3^ School of Engineering Sciences in Chemistry, Biotechnology and Health, KTH Royal Institute of Technology, Huddinge, Sweden

^4^ IMM Institute of Environmental Medicine, Karolinska Institutet, Stockholm, Sweden

**Corresponding author:** Suzanne L. Merkus**,** National Institute of Occupational Health, Pb 5330 Majorstuen, 0304 Oslo, Norway**.** E-mail: [suzanne.merkus@stami.no](mailto:suzanne.merkus@stami.no). Phone: (+47) 2319 5100. [www.stami.no](http://www.stami.no)

## Journal

International Archives of Occupational and Environmental Health

## Appendix C. Figures of the isotemporal substitution models

*
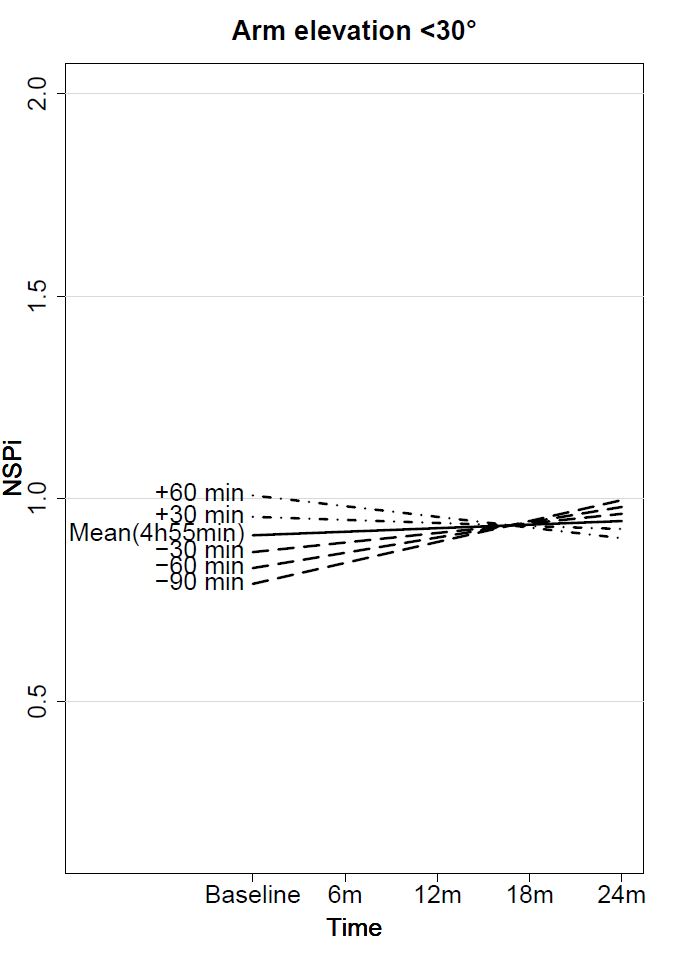

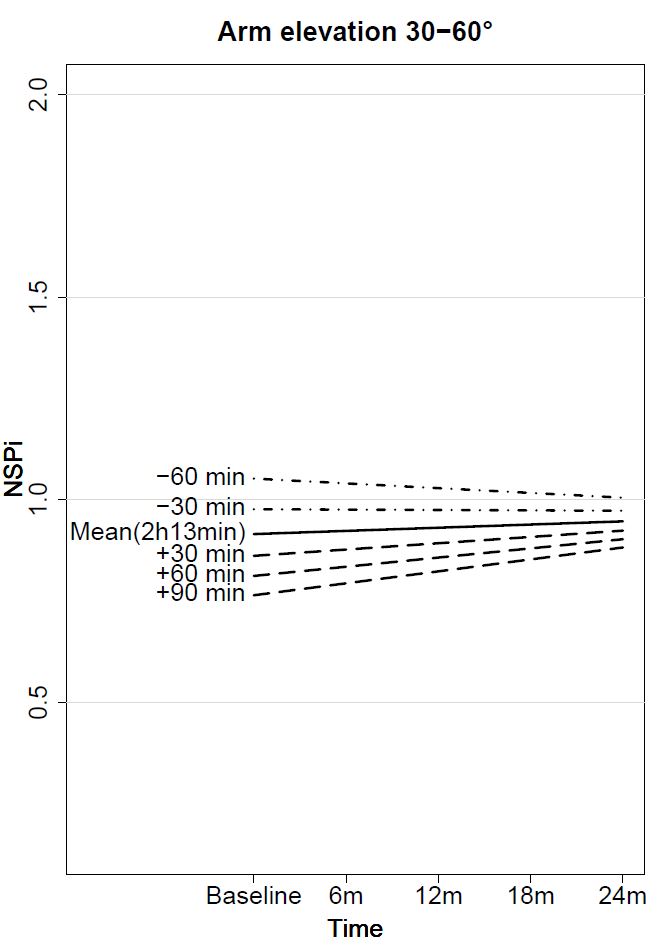

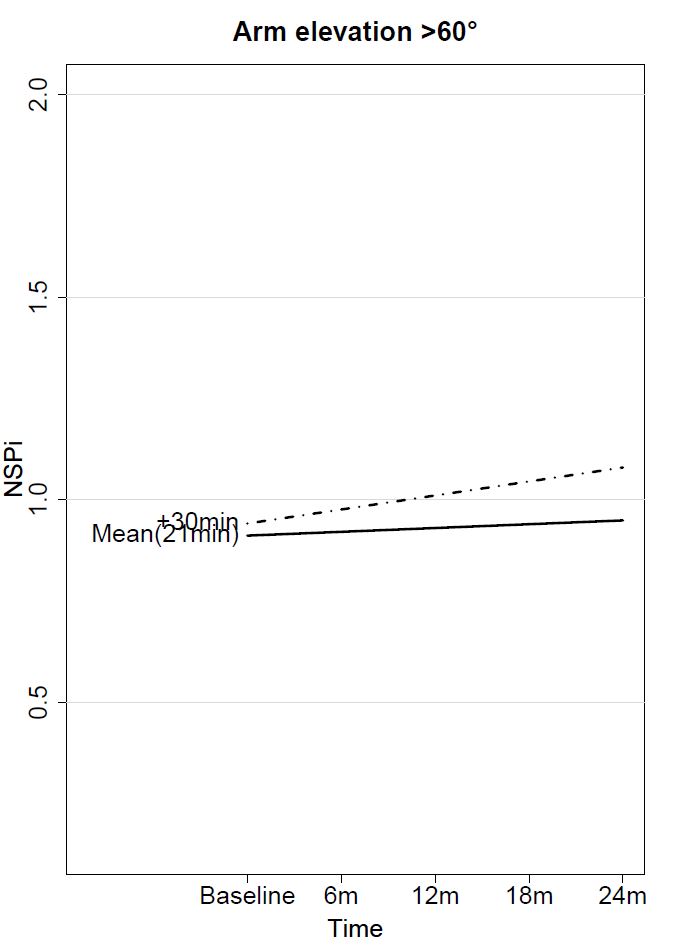
*

*Figure C.1. Estimated average change in NSPi (on a scale from 0-3) during a 2-year follow-up when reallocating time in 30 minute increments from and to time spent in upper arm elevation <30°, 30-60°, >60° (one-to-all reallocation). NB, for reader clarity, the y-axis ranges from 0-2 and thereby represents part of the full 0-3 NSPi scale.*


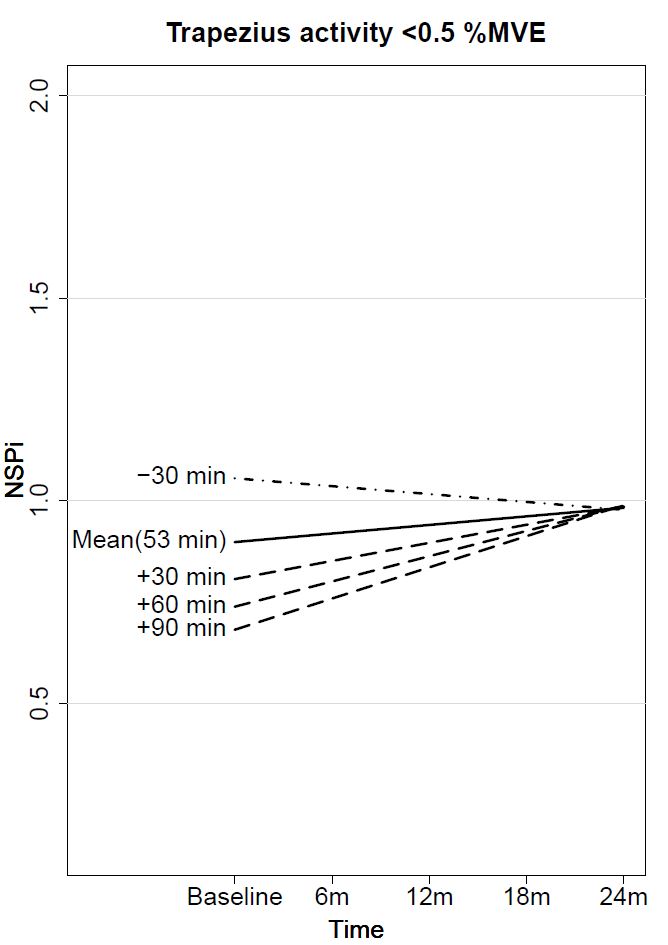

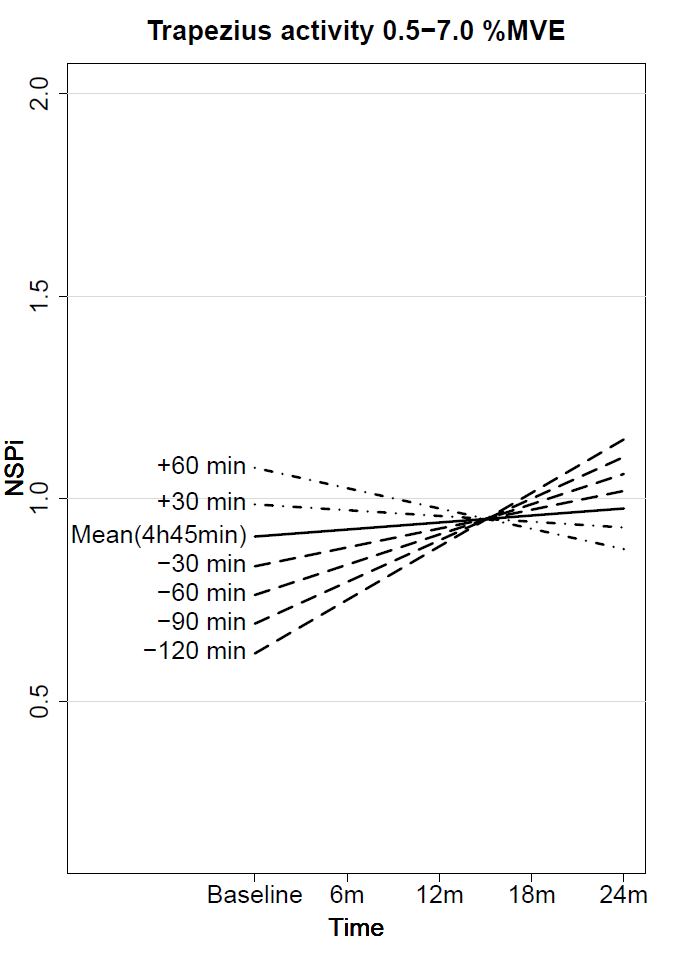

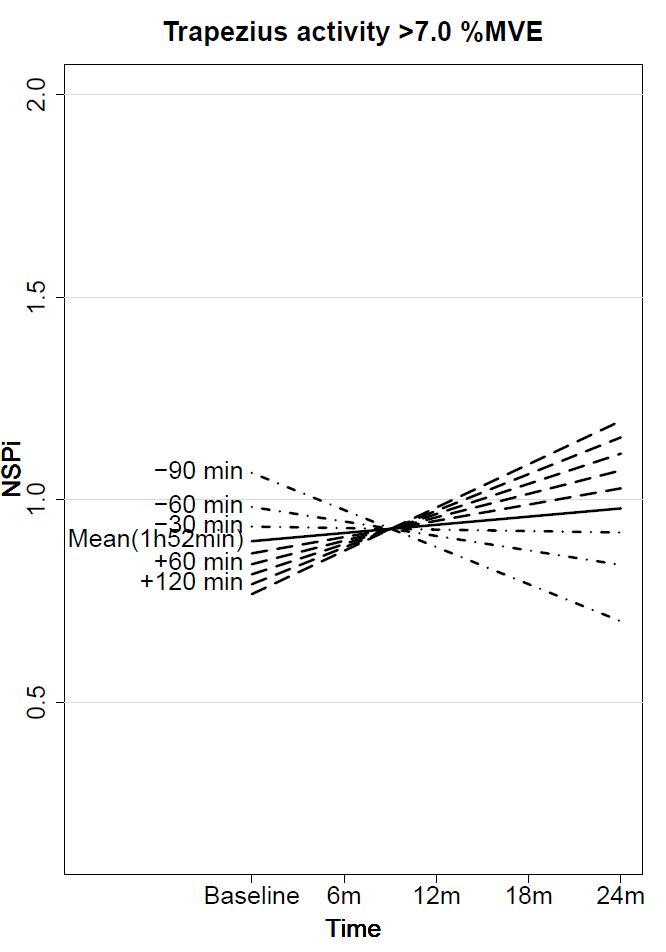


*Figure C.2. Estimated average change in NSPi (on a scale from 0-3) during a 2-year follow-up when reallocating time in 30 minutes increments from and to time spent in upper trapezius muscle activity <0.5 %MVE, 0.5-7.0 %MVE, and >7.0 %MVE (one-to-all reallocation). NB, for reader clarity, the y-axis ranges from 0-2 and thereby represents part of the full 0-3 NSPi scale.*


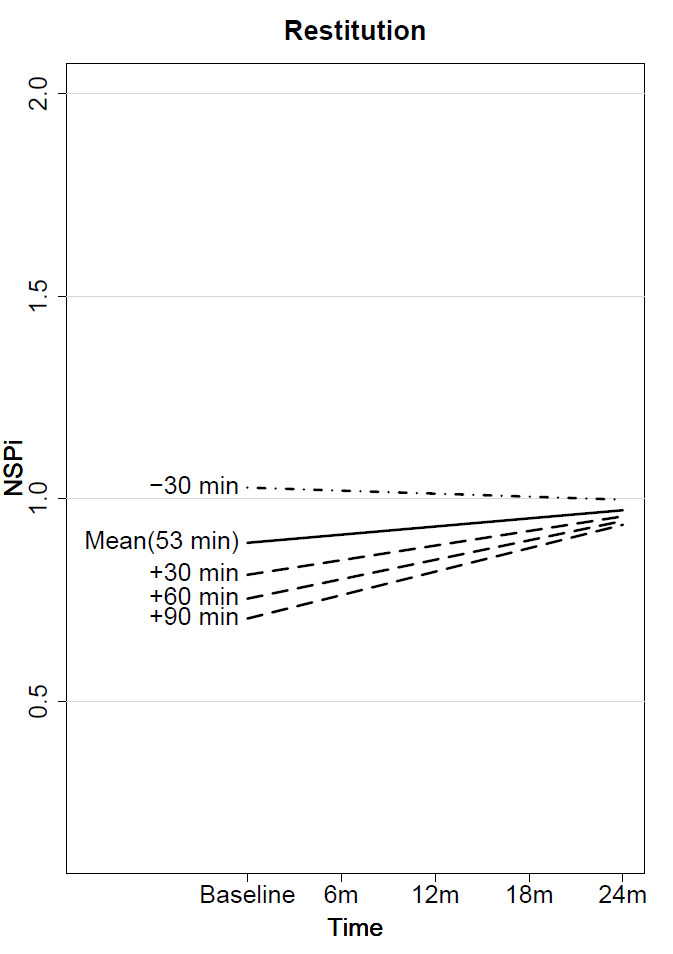

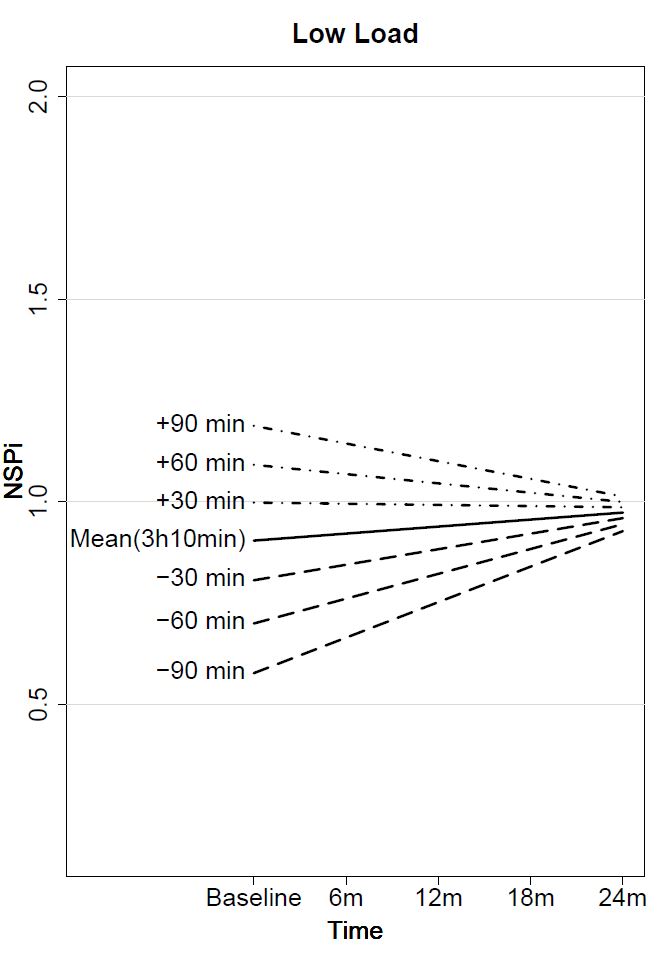


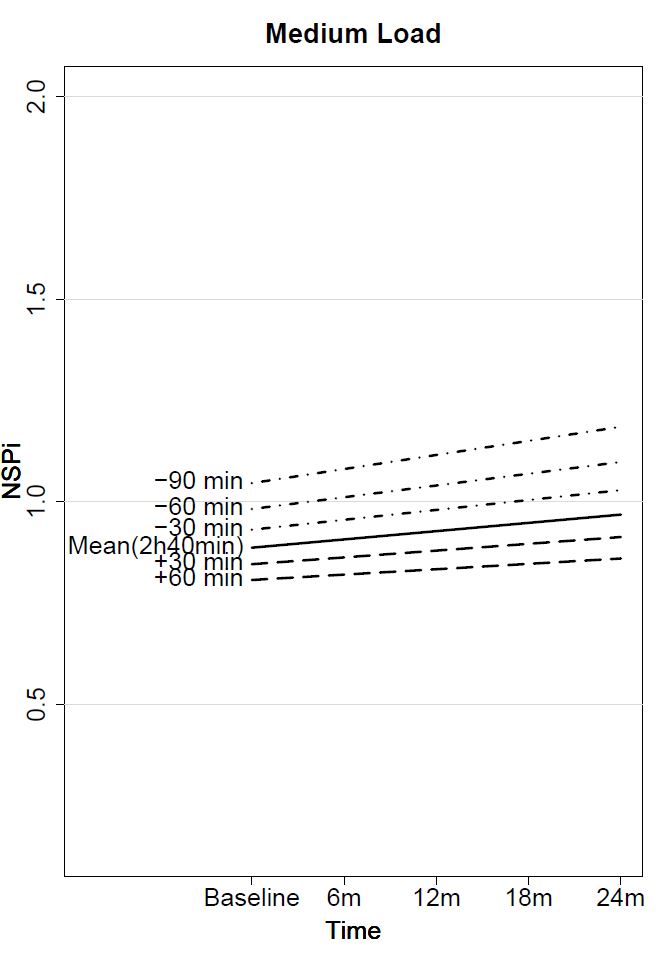

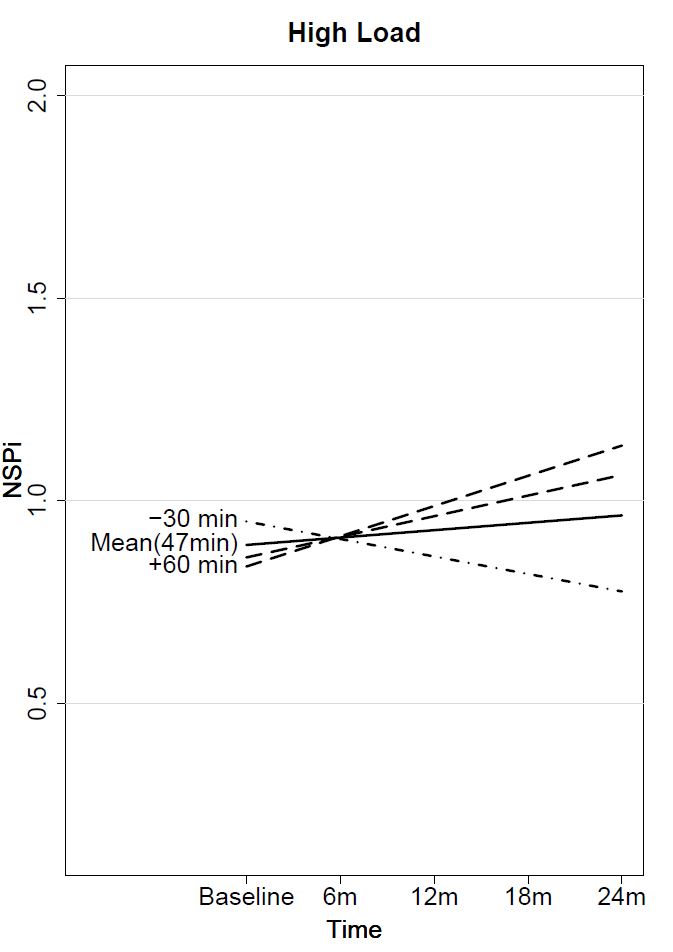


*Figure C.3. Estimated average change in NSPi (on a scale from 0-3) during a 2-year follow-up when reallocating time in 30 minutes increments to and from time spent in restitution, low, medium, and high neck/shoulder load (one-to-all reallocation). NB, for reader clarity, the y-axis ranges from 0-2 and thereby represents part of the full 0-3 NSPi scale*
