# Supplementary material for: Can a metric combining arm elevation and trapezius muscle activity predict neck/shoulder pain? A prospective cohort study in construction and healthcare
Source: Int Arch Occup Environ Health. 2020 Dec 5;94(4):647–58. doi: 10.1007/s00420-020-01610-w (PMC8068682; doi:10.1007/s00420-020-01610-w)
Supplement: Supplementary file 4 — Supplementary file4 (DOCX 26 KB) [file 420_2020_1610_MOESM4_ESM.docx]

# **Can a metric combining arm elevation and trapezius muscle activity predict neck/shoulder pain? A prospective cohort study in construction and healthcare**

Suzanne Lerato Merkus^1^ (ORCID 0000-0003-0945-3738)

Svend Erik Mathiassen (ORCID 0000-0003-1443-6211)^2^

Lars-Kristian Lunde (ORCID 0000-0001-6219-9244) ^1^

Markus Koch (PhD) ^1^

Morten Wærsted (ORCID 0000-0002-9570-2181) ^1^

Mikael Forsman (ORCID 0000-0001-5777-4232)^3,4^

Stein Knardahl (ORCID 0000-0002-7300-8519) ^1^

Kaj Bo Veiersted (ORCID 0000-0003-1221-384X)^1^

^1^ National Institute of Occupational Health, Oslo, Norway

^2^ Centre for Musculoskeletal Research, Department of Occupational and Public Health Sciences, University of Gävle, Gävle, Sweden

^3^ School of Engineering Sciences in Chemistry, Biotechnology and Health, KTH Royal Institute of Technology, Huddinge, Sweden

^4^ IMM Institute of Environmental Medicine, Karolinska Institutet, Stockholm, Sweden

**Corresponding author:** Suzanne L. Merkus**,** National Institute of Occupational Health, Pb 5330 Majorstuen, 0304 Oslo, Norway**.** E-mail: [suzanne.merkus@stami.no](mailto:suzanne.merkus@stami.no). Phone: (+47) 2319 5100. [www.stami.no](http://www.stami.no)

## Journal

International Archives of Occupational and Environmental Health

## Appendix D. Sensitivity analyses

**Table D.1. Crude and adjusted models for the association of the course of neck/shoulder pain with durations of upper arm elevation (n=45), upper trapezius muscle activity (n=34), and neck/shoulder load (n=34) for those without pain at baseline**

|  | **Crude model** | | |  | **Adjusted model^a^** | | |
| --- | --- | --- | --- | --- | --- | --- | --- |
| **Arm elevation (n=45)** | **ß** | **SE** | **p-value** |  | **ß** | **SE** | **p-value** |
| Time | **0.17** | **0.08** | **0.024** |  | **0.18** | **0.08** | **0.021** |
| <30° (vs >30°) | -0.02 | 0.10 | 0.867 |  | 0.03 | 0.10 | 0.755 |
| *<30° (vs >30°)*Time* | -0.03 | 0.04 | 0.563 |  | 0.05 | 0.04 | 0.193 |
| 30-60° (vs <30° & >60°) | 0.00 | 0.12 | 0.992 |  | -0.03 | 0.12 | 0.794 |
| *30-60° (vs <30° & >60°)*Time* | -0.03 | 0.05 | 0.608 |  | -0.03 | 0.05 | 0.602 |
| >60° (vs <60°) | 0.02 | 0.10 | 0.871 |  | 0.00 | 0.11 | 0.999 |
| >*60° (vs <60°)*Time* | 0.05 | 0.04 | 0.213 |  | -0.03 | 0.04 | 0.539 |
|  | **Crude model** | | |  | **Adjusted model^b^** | | |
| **Muscle activity (n=34)** | **ß** | **SE** | **p-value** |  | **ß** | **SE** | **p-value** |
| Time | 0.16 | 0.12 | 0.503 |  | 0.37 | 0.24 | 0.123 |
| <0.5% MVE (vs >0.5% MVE) | -0.08 | 0.12 | 0.503 |  | -0.08 | 0.11 | 0.472 |
| *<0.5% MVE (vs >0.5% MVE)*Time* | 0.02 | 0.05 | 0.682 |  | 0.03 | 0.05 | 0.552 |
| 0.5-6.9 % MVE (vs <0.5% & >7.0% MVE) | 0.11 | 0.21 | 0.596 |  | 0.10 | 0.22 | 0.636 |
| *0.5-6.9% MVE (vs <0.5% & >7.0%* *MVE)*Time* | -0.06 | 0.10 | 0.561 |  | -0.04 | 0.10 | 0.669 |
| >7.0% MVE (vs <7.0% MVE) | -0.03 | 0.14 | 0.821 |  | -0.02 | 0.16 | 0.897 |
| *>7.0% MVE (vs <7.0% MVE)*Time* | 0.04 | 0.06 | 0.584 |  | 0.01 | 0.07 | 0.851 |
|  | **Crude model** | | |  | **Adjusted model^c^** | | |
| **Neck/shoulder load (n=34)** | **ß** | **SE** | **p-value** |  | **ß** | **SE** | **p-value** |
| Time | 0.26 | 0.08 | 0.002 |  | 0.43 | 0.22 | 0.055 |
| Restitution (vs shoulder load) | -0.05 | 0.09 | 0.562 |  | -0.05 | 0.09 | 0.560 |
| *Restitution (vs shoulder load)*Time* | 0.03 | 0.04 | 0.472 |  | 0.04 | 0.04 | 0.335 |
| Low load (vs restitution, medium, high load) | 0.04 | 0.16 | 0.801 |  | 0.08 | 0.16 | 0.641 |
| Low load (vs restitution, medium, high load)**Time* | 0.05 | 0.07 | 0.442 |  | 0.05 | 0.07 | 0.507 |
| Medium load (vs restitution, low, high load) | -0.05 | 0.23 | 0.837 |  | -0.08 | 0.26 | 0.762 |
| *Medium load (vs restitution, low, high load)*Time* | -0.21 | 0.11 | 0.050 |  | **-0.23** | **0.11** | **0.039** |
| High load (vs restitution, low, medium load) | 0.06 | 0.15 | 0.671 |  | 0.05 | 0.17 | 0.745 |
| *High load (vs restitution, low, medium load)*Time* | **0.13** | **0.06** | **0.038** |  | **0.15** | **0.07** | **0.031** |
| **Bold face mark associations with p<0.05*  *^a^ Adjusted for gender, sector, and NSP duration in the 12 months preceding baseline*  *^b^ Adjusted for gender, sector, NSP duration in the 12 months preceding baseline, social climate, social climate*time, control of work pacing, control of work pacing*time*  ***^c^*** *Adjusted for sector and NSP duration in the 12 months preceding baseline, social climate, social climate*time, control of work pacing, control of work pacing*time* | | | | | | | |

**Table D.2. Crude and adjusted models for the association of the course of neck/shoulder pain with durations of upper arm elevation (n=69), upper trapezius muscle activity (n=56), and neck/shoulder load (n=53) for those with pain at baseline**

|  | **Crude model** | | | |  | **Adjusted model^a^** | | |
| --- | --- | --- | --- | --- | --- | --- | --- | --- |
| **Arm elevation (n=69)** | **ß** | | **SE** | **p-value** |  | **ß** | **SE** | **p-value** |
| Time | 0.01 | | 0.09 | 0.877 |  | 0.004 | 0.09 | 0.965 |
| <30° (vs >30°) | 0.19 | | 0.21 | 0.381 |  | 0.08 | 0.20 | 0.707 |
| *<30° (vs >30°)*Time* | -0.11 | | 0.07 | 0.100 |  | -0.11 | 0.07 | 0.107 |
| 30-60° (vs <30° & >60°) | -0.17 | | 0.29 | 0.559 |  | -0.10 | 0.27 | 0.715 |
| *30-60° (vs <30° & >60°)*Time* | 0.12 | | 0.09 | 0.167 |  | 0.13 | 0.09 | 0.159 |
| >60° (vs <60°) | -0.02 | | 0.15 | 0.904 |  | 0.02 | 0.15 | 0.880 |
| >*60° (vs <60°)*Time* | -0.01 | | 0.05 | 0.780 |  | -0.02 | 0.05 | 0.708 |
|  | **Crude model** | | | |  | **Adjusted model^b^** | | |
| **Muscle activity (n=56)** | **ß** | **SE** | | **p-value** |  | **ß** | **SE** | **p-value** |
| Time | 0.03 | 0.09 | | 0.728 |  | **0.42** | **0.20** | **0.036** |
| <0.5% MVE (vs >0.5% MVE) | 0.00 | 0.13 | | 0.990 |  | -0.02 | 0.13 | 0.877 |
| *<0.5% MVE (vs >0.5% MVE)*Time* | 0.00 | 0.05 | | 0.972 |  | 0.01 | 0.05 | 0.768 |
| 0.5-6.9 % MVE (vs <0.5% & >7.0% MVE) | -0.05 | 0.23 | | 0.813 |  | 0.03 | 0.22 | 0.887 |
| *0.5-6.9% MVE (vs <0.5% & >7.0%* *MVE)*Time* | -0.10 | 0.08 | | 0.199 |  | -0.09 | 0.08 | 0.240 |
| >7.0% MVE (vs <7.0% MVE) | 0.06 | 0.14 | | 0.684 |  | -0.01 | 0.13 | 0.936 |
| *>7.0% MVE (vs <7.0% MVE)*Time* | **0.10** | **0.05** | | **0.041** |  | 0.08 | 0.05 | 0.118 |
|  | **Crude model** | | | |  | **Adjusted model^c^** | | |
| **Neck/shoulder load (n=53)** | **ß** | **SE** | | **p-value** |  | **ß** | **SE** | **p-value** |
| Time | 0.01 | 0.13 | | 0.922 |  | 0.35 | 0.22 | 0.109 |
| Restitution (vs shoulder load) | -0.04 | 0.12 | | 0.763 |  | -0.01 | 0.12 | 0.947 |
| *Restitution (vs shoulder load)*Time* | -0.01 | 0.04 | | 0.725 |  | -0.01 | 0.04 | 0.829 |
| Low load (vs restitution, medium, high load) | -0.03 | 0.27 | | 0.921 |  | 0.01 | 0.27 | 0.961 |
| Low load (vs restitution, medium, high load)**Time* | -0.10 | 0.09 | | 0.244 |  | -0.09 | 0.09 | 0.286 |
| Medium load (vs restitution, low, high load) | 0.21 | 0.38 | | 0.580 |  | 0.11 | 0.37 | 0.760 |
| *Medium load (vs restitution, low, high load)*Time* | 0.07 | 0.16 | | 0.652 |  | 0.11 | 0.16 | 0.473 |
| High load (vs restitution, low, medium load) | -0.15 | 0.24 | | 0.545 |  | -0.12 | 0.23 | 0.610 |
| *High load (vs restitution, low, medium load)*Time* | 0.05 | 0.10 | | 0.649 |  | -0.01 | 0.10 | 0.935 |
| **Bold face mark associations with p<0.05*  *^a^ Adjusted for gender, sector, and NSP duration in the 12 months preceding baseline*  *^b^ Adjusted for gender, sector, NSP duration in the 12 months preceding baseline, social climate, social climate*time, control of work pacing, control of work pacing*time*  **^c^** *Adjusted for sector and NSP duration in the 12 months preceding baseline, social climate, social climate*time, control of work pacing, control of work pacing*time* | | | | | | | | |
